# A Leaderless Genome Identified during Persistent Bovine Coronavirus Infection Is Associated with Attenuation of Gene Expression

**DOI:** 10.1371/journal.pone.0082176

**Published:** 2013-12-12

**Authors:** Ting-Yung Ke, Wei-Yu Liao, Hung-Yi Wu

**Affiliations:** Institute of Pathobiology, College of Veterinary Medicine, National Chung-Hsing University, Taichung, Taiwan ROC; Kantonal Hospital St. Gallen, Switzerland

## Abstract

The establishment of persistent viral infection is often associated with the selection of one or more mutant viruses. For example, it has been found that an intraleader open reading frame (ORF) in genomic and subgenomic mRNA (sgmRNA) molecules is selected during bovine coronavirus (BCoV) persistence which leads to translation attenuation of the downstream ORF. Here, we report the unexpected identification of leaderless genomes, in addition to leader-containing genomes, in a cell culture persistently infected with BCoV. The discovery was made by using a head-to-tail ligation method that examines genomic 5′-terminal sequences at different times postinfection. Functional analyses of the leaderless genomic RNA in a BCoV defective interfering (DI) RNA revealed that (1) the leaderless genome was able to serve as a template for the synthesis of negative-strand genome, although it cannot perform replicative positive-strand genomic RNA synthesis, and (2) the leaderless genome retained its function in translation and transcription, although the efficiency of these processes was impaired. Therefore, this previously unidentified leaderless genome is associated with the attenuation of genome expression. Whether the leaderless genome contributes to the establishment of persistent infection remains to be determined.

## Introduction

The families Coronaviridae (Coronavirus and Torovirus genera) and Arteriviridae, together with Roniviridae, are members of order Nidovirales in which a nested set of subgenomic RNA (sgmRNA) molecules are made that are 3′-coterminal with the genome during transcription [1]–[6]. In arteriviruses and coronaviruses, sgmRNAs are both 3′-coterminal and contain a common 5′ leader sequence derived from the 5′ end of the genome [7]–[9]. However, all sgmRNAs in gill-associated virus (GAV), and sgmRNAs 3, 4, and 5 in equine torovirus (EToV), lack a leader sequence identical to the 5′ end of the genome [5]–[6]. The mechanism of leader acquisition during discontinuous negative-strand ((−)-strand) synthesis from the positive-strand ((+)-strand) genomic RNA template has gained favor to explain how sgmRNAs acquire a leader sequence from the 5′ end of the genome [9]–[15]. The mechanism has been applied to explain high-frequency leader-switching events during the replication of the coronavirus defective interfering (DI) RNA genome [16]–[19].

To date, the function of a leader sequence in the genome or subgenome during the coronavirus life cycle has not been systematically established. It has been suggested that the leader sequence within the context of the mouse hepatitis virus (MHV) DI RNA containing the chloramphenicol acetyltransferase (CAT) gene is nonessential for negative-strand DI RNA synthesis [20], whereas for replication (interpreted as positive-strand RNA synthesis) of bovine coronavirus (BCoV) DI RNA the leader is thought to be required [19]. A 25–59-nucleotide (nt) sequence motif within the leader sequence has been demonstrated to be required for transcription using MHV DI RNA with CAT gene [21]. Although replacement of the α-globin mRNA 5′ UTR with the MHV leader sequence has been suggested to enhance the translation of α-globin mRNA [22], the role of the leader sequence in genome translation remains unknown.

Structural changes within the leader sequence of BCoV have been linked to persistent infection in cell culture due to the selection of a translation-attenuating intraleader open reading frame (ORF) [23]. In MHV, 5′ UTR changes have also been found to arise during persistent infection in cell culture [24] but in this case, a mutation was found downstream of the leader that caused an enhancement of gene expression rather than attenuation of translation. The mechanisms that establish persistent infections in coronaviruses are still not understood; however, as with other RNA viruses, the interplay between virus and host may be the key to initiate persistence, which is then followed by the selection of a mutation to maintain the persistent infection [25]–[28]. Although there are some exceptions [24], 28–29,30, the general principle for establishment of virus persistence appears to be the attenuation virus gene expression and a restriction of cytopathic effect [25], [29], [31]. Whether there are mutations other than the one described above that might contribute to BCoV persistent infection remains to be determined.

In a study designed to identify potential sequence heterogeneity within the 5′ end of genomic RNA during BCoV persistent infection, we unexpectedly found a leaderless genome in addition to the leader-containing genome. Along with evidence in previous studies [32]–[36], this finding suggests that viruses in the order Nidovirales may be evolutionarily derived from a common leaderless ancestor since leaderless sgmRNAs are also identified in other nidoviruses as a normal condition. Functional analyses of BCoV DI RNA suggested that the leaderless genome is able to attenuate translation, negative-strand synthesis, and transcription. Therefore, the leaderless genome identified during BCoV persistence appears associated with the attenuation of gene expression. A proposed consequence of the leaderless genome in persistent infection is discussed.

## Results

### Identification of a Leaderless Genomic RNA during Bovine Coronavirus Persistent Infection

**Figure 1:**
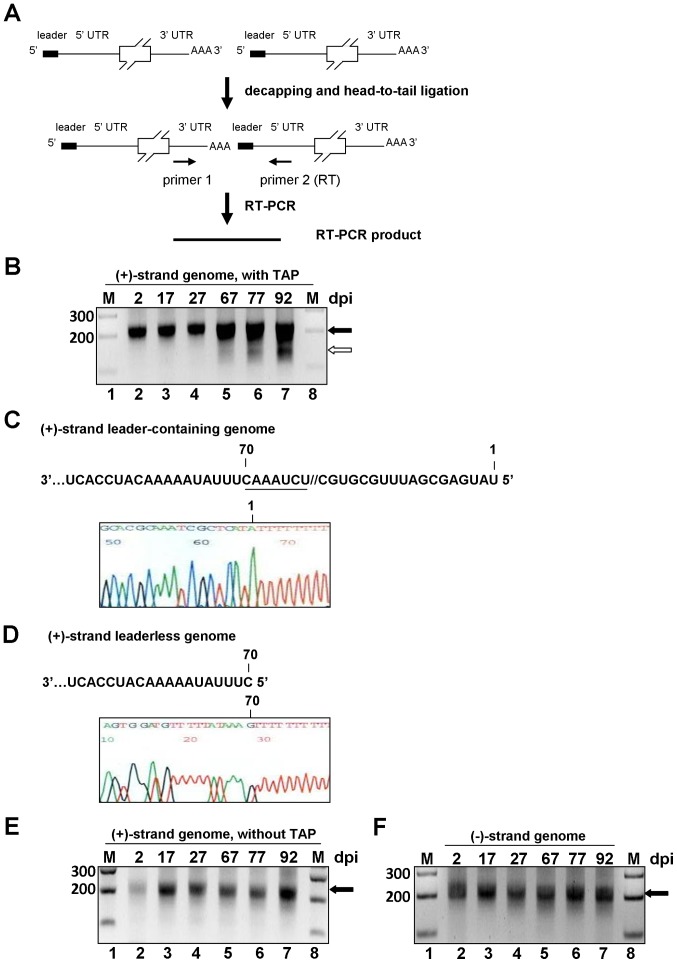
Identification of leaderless genomic RNA during BCoV persistent infection. (A) Strategy to identify positive-strand leaderless genomic RNA. Poly(A)-containing RNA was selected from total cellular RNA extracted from BCoV-persistently infected cells, treated with alkaline phosphatase, decapped with tobacco acid pyrophosphatase, head-to-tail ligated with T4 RNA ligase I, and used as the template for RT-PCR with the BCoV 5′ UTR-(+)-strand-specific primer 2: BCV107(+) (for RT) and BCoV 3′ UTR-(−)-strand-specific primer 1: BCV3′UTR1(−). (B) RT-PCR product synthesized by the method described in Fig. 1A. RT-PCR products with a size of more than 200 bp (lanes 2–7, marked with black arrowhead) and with a size of less than 200 bp (lanes 6–7, marked with white arrowhead) were observed. (C) The upper panel shows part of the first 88-nt sequence of the 5′ UTR in the positive-strand BCoV genomic RNA. The positions (1 and 70) are given on the top of the sequence, and the intergenic sequence (IS) UCUAAAC is underlined. The lower panel shows the sequence (shown in the negative strand) of the cDNA-cloned RT-PCR product with a size of more than 200 bp from lane 7, as indicated with a black arrowhead in Fig. 1B. (D) The upper panel shows the sequence of the 5′UTR on the positive-strand BCoV genomic RNA, which lacks the first 69 nts; position 70 is given on the top of the sequence. The lower panel shows the sequence (shown in the negative strand) of the cDNA-cloned RT-PCR product with a size of less than 200 bp from lane 7, as indicated with a white arrowhead in Fig. 1B. (E) Control reactions to determine if the positive-strand leaderless genome is a degradation product. RT-PCR product was synthesized by the method described in Fig. 1A except RNA sample was not treated with alkaline phosphatase and tobacco acid pyrophosphatase. RT-PCR products with a length of more than 200 bp were detected. (F) Identification of negative-strand leaderless genomic RNA. Total cellular RNA was treated with tobacco acid pyrophosphatase and ligated with T4 RNA ligase I. RT-PCR product was synthesized by the method described in Fig. 1A except that primer BCV3′UTR1(−) was used for RT. RT-PCR products with a size of more than 200 bp (lanes 2–7, marked with black arrowhead) were observed. M, ds DNA size markers in nt pairs. dpi: days postinfection.

In an attempt to identify the 5′-terminal sequence of the positive-strand viral genomic RNA during BCoV persistent infection in cell culture, total cellular RNA was extracted at the time points indicated in Fig. 1B and the RNA population containing a poly(A) tail was selected. The prepared poly(A)-containing RNA was then treated with alkaline phosphatase, decapped with tobacco acid pyrophosphatase and head-to-tail ligated with T4 RNA ligase, followed by RT-PCR and sequencing (Fig. 1A). With the use of a PCR primer set that specifically anneals within the 5′ and 3′ UTRs of the BCoV genome, RT-PCR products with a length of more than 200 base pairs (bp) were observed at 2, 17, 27, and 67 days postinfection (dpi) (Fig. 1B, lanes 2–5). However, two species of RT-PCR products were produced from RNA extracted at 77 and 92 dpi that were not present earlier: the major product with a length larger than 200 bp and the minor one with a length smaller than 200 bp (Fig. 1B, lanes 6 and 7). The concentration ratio of the major product to the minor product was roughly 5 to 1. To determine more rigorously whether the leaderless genome was present at earlier time points, a seminested PCR was performed at all time points but again no RT-PCR product showing a leaderless genome was found at the earlier time points (data not shown). Sequencing of the two RT-PCR products (Fig. 1B, lanes 6 and 7) revealed that the major band (>200 bp) had an intact positive-strand 5′-terminal 65-nt leader sequence (Fig. 1C), whereas the minor band (<200 bp) lacked the 5′-terminal 69 nt (Fig. 1D). To determine whether the identified RNA molecule with 5′-terminal 69-nt deletion is a degraded product, total cellular RNA was extracted, poly(A) tail-selected, and head-to-tail ligated with T4 RNA ligase, but not treated with alkaline phosphatase and tobacco acid pyrophosphatase followed by RT-PCR. As shown in Fig. 1E, an RT-PCR product with a length greater than 200 bp was detected, indicating that there is still some degraded leader-containing RNA in the infected cells. However, an RT-PCR product of less than 200 bp was not observed, indicating that the 5′-terminal 69 nt-deleted RNA molecule was not an artifact resulting from the cleavage of the positive-strand leader-containing genomic RNA as observed in Fig. 1B. To test whether a leaderless negative-strand genomic RNA is also produced during BCoV persistence, the same primers were used as for identification of positive-strand genomic RNA, but primer 1: BCV3′UTR1(−) was used for the RT. However, only RT-PCR products with a length of more than 200 bp were observed (Fig. 1F, lanes 2–7). We speculated that if there are leaderless negative-strand genomic RNAs in persistently-infected cells, they may be present in numbers too low to be detected by RT-PCR. In BCoV persistently infected cells, by Northern analysis at 76 day of persistent infection, there are an estimated ∼10 and ∼500 molecules of genome and sgmRNA 7 per cell, respectively, and ∼0 and ∼10 molecules of genome negative strand and sgmRNA 7 negative strand per cell, respectively [37]. Therefore we chose to look for leaderless negative-strand sgmRNA 7 in persistent BCoV-infected HRT-18 cells. As shown in Fig. S1A, lanes 5–7, two species of RT-PCR products were produced from RNA extracted at 67, 77 and 92 dpi. Sequencing of the two RT-PCR products (Fig. S1A, lanes 5–7) revealed that the product with the longer length had an intact negative-strand 3′-terminal sequence (Fig. S1B), whereas the RT-PCR product with the shorter length lacked the 3′-terminal 69 nt (Fig. S1C). These results demonstrate that the leaderless negative-strand sgmRNA 7 is also synthesized during persistent BCoV infection and may explain why the very low abundance of leaderless negative-strand genome is not detected. Taken together, these results suggest that both the leader-containing and leaderless positive-strand genomic RNA molecules coexist during BCoV persistent infection in cell culture.

### DI RNA with a Leader Sequence Translates with Significantly Greater Efficiency than DI RNA without a Leader Sequence in BCoV-infected Cells

**Figure 2:**
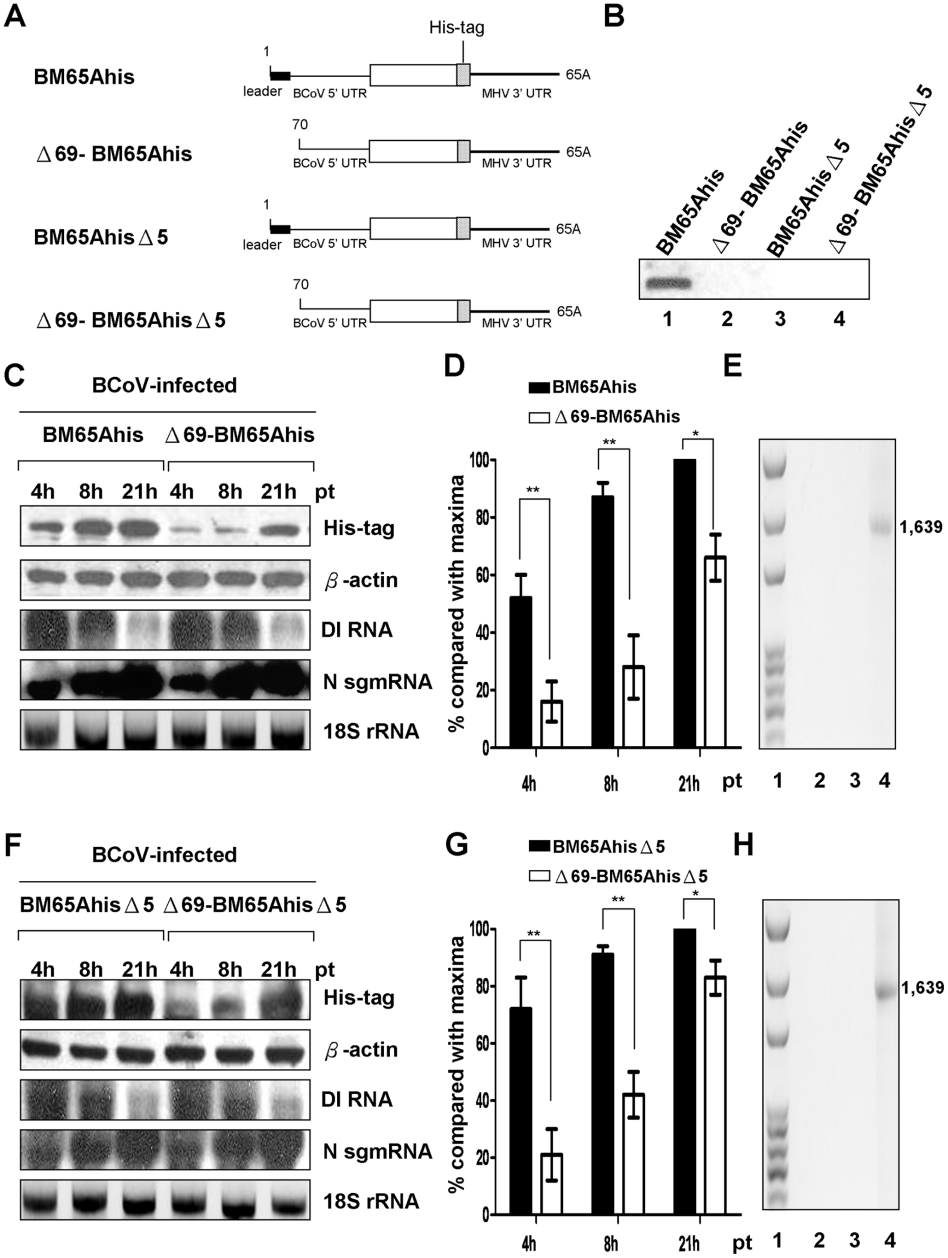
Effect of leaderless DI RNA on translation. (A) DI RNA constructs with a His-tag used for replication and translation assay. Each DI RNA construct has an open reading frame (open box), followed by an in-frame 18-nt His-tag coding region (stippled box) and MHV 3′ UTR. (B) Replication of DI RNA by Northern blot assay. RNA samples collected at 48 hpi of VP1 were used to determine the replication of the DI RNA. (C) and (F) Protein expression from the DI RNA constructs. BCoV-infected HRT-18 cells were transfected with the indicated DI RNA construct at 2 hpi, and total intracellular proteins or RNA was extracted at 4, 8, and 21 hpt for analysis. Western blotting was used to measure the abundance of His-tagged protein and β-actin. The levels of DI RNA, N sgmRNA, and 18S rRNA were measured by Northern blotting. (D) and (G) Quantitation analysis of the His-tagged protein from individual DI RNA constructs at different time points. (E) and (H) RT-PCR to detect a potential recombinant between the BCoV genome and DI RNA. The primers MHV3′UTR2(+), which anneal to the MHV 3′ UTR and was used for RT, and M3(−), which anneal to the BCoV M protein gene, were used for PCR to detect potential recombination between the BCoV genome and BM65Ahis (Fig. 2E, lane 2), Δ69-BM65Ahis (Fig. 2E, lane 3), BM65AhisΔ5 (Fig. 2H, lane 2), or Δ69-BM65AhisΔ5 (Fig. 2H, lane 3). The recombinant DNA of 1,639 nt shown in lane 4 of Figs. 2E and 2H was generated by overlap RT-PCR and was used as a size marker for the product generated using the primers MHV 3′ UTR2(+) and M3(−). The values (D) and (G) represent the mean±SEM of three individual experiments. *p<0.05, **p<0.01.

All the viruses in the order Nidovirales contain a leader sequence at the 5′ end of the positive-strand genome [15]. The identification of a previously unnoticed leaderless genomic RNA during BCoV persistent infection raises a question with regard to the biological significance of the leaderless genome. The replacement of the α-globin mRNA 5′ UTR with the MHV 5′-leader sequence has been suggested to cause an increase of the translation efficiency for α-globin mRNA, indicating a probable requirement of a leader sequence in sgmRNA translation [22]. Based on this finding, we speculated that one of the functions of the leaderless genomic RNA may be to downregulate viral translation. To examine this possibility, we used BCoV DI RNA, a naturally occurring DI RNA that has been employed as a surrogate for the BCoV genome to analyze *cis*-acting elements required for replication and translation [38]–[44]. To test the effect of the leaderless genome on translation, BCoV DI RNAs BM65Ahis (with the leader sequence) and Δ69-BM65Ahis (without the leader sequence) containing the MHV 3′ UTR were constructed [45]. In addition, to specifically define the function of the leader sequence in coronavirus translation without the influence of replication, replication-incompetent DI RNAs BM65AhisΔ5 (with the leader sequence) and Δ69-BM65AhisΔ5 (without the leader sequence) in which the last 5 nt of the 3′ UTR of both constructs were deleted were also generated and tested (Figs. 2A and 2B). Histidine residues were used as a tag in both constructs to test the translation efficiency in the BCoV-infected cells by Western blot analysis. Antibody specific to the histidine tag was used. The His-tagged proteins expressed from BM65Ahis and Δ69-BM65Ahis are shown in Fig. 2C, and a quantitation analysis of the translation products (Fig. 2D) revealed that the expressed proteins from both constructs increased over time. However, the translation efficiency from Δ69-BM65Ahis was ∼3- and ∼2-fold less compared to that from BM65Ahis at 4 and 8 hours posttransfection (hpt) (p<0.01), respectively, although the translation efficiency between the two constructs became closer at 21 hpt (66% vs 100%, p<0.05). Because recombination between the input DI RNA and coronavirus genome may occur under certain selection pressures [46]–[48], the detected proteins may be translated from a recombinant containing the coronavirus genome and DI RNA genes. To ensure the expressed His-tagged protein arose specifically from DI RNA and not from a recombinant molecule, RT-PCR using primers that anneal to the reporter sequence in DI RNA (for RT) and the M protein gene in the coronavirus genome was used to test for a potential recombinant generated during infection [49]. No RT-PCR product was observed (Fig. 2E, lanes 2–3), thus ruling out the expression of the protein from a recombinant molecule. These results suggested that the translation of leaderless DI RNA is less efficient than that of DI RNA with a leader during infection.

In addition to the present study (Fig. 2B, lanes 1–2), it has been demonstrated that the leader sequence is required for DI RNA replication [19]. To exclude the effect of replication on translation and to specifically define the role of the leader sequence in translation, replication-blocked DI constructs BM65AhisΔ5 (with the leader sequence) and Δ69-BM65AhisΔ5 (without the leader sequence) (Figs. 2A and 2B) were generated and examined in BCoV-infected HRT-18 cells. The His-tagged proteins expressed from both DI RNA constructs were also detected over time (Fig. 2F). After quantitation (Fig. 2G), the translation efficiency from Δ69-BM65AhisΔ5 was ∼3- and ∼2-fold less at 4 and 8 hpt (p<0.01), respectively, when compared to that from BM65AhisΔ5, whereas the translation efficiency between the two constructs was closer at 21 hpt (85% vs 100%, p<0.05). The same primers described above (Fig. 2E) were also employed to identify potential recombination between DI RNA and the virus genome, and no RT-PCR product was detected (Fig. 2H, lanes 2–3). These results suggest that one of the functions of the leader sequence in the coronavirus genome is to enhance translation. Taken together, it appears that translation is decreased during BCoV infection as a result of the lower translation efficiency of leaderless genomic RNA.

### The Leader Sequence Enhances the Efficiency of Negative-strand DI RNA Synthesis

**Figure 3:**
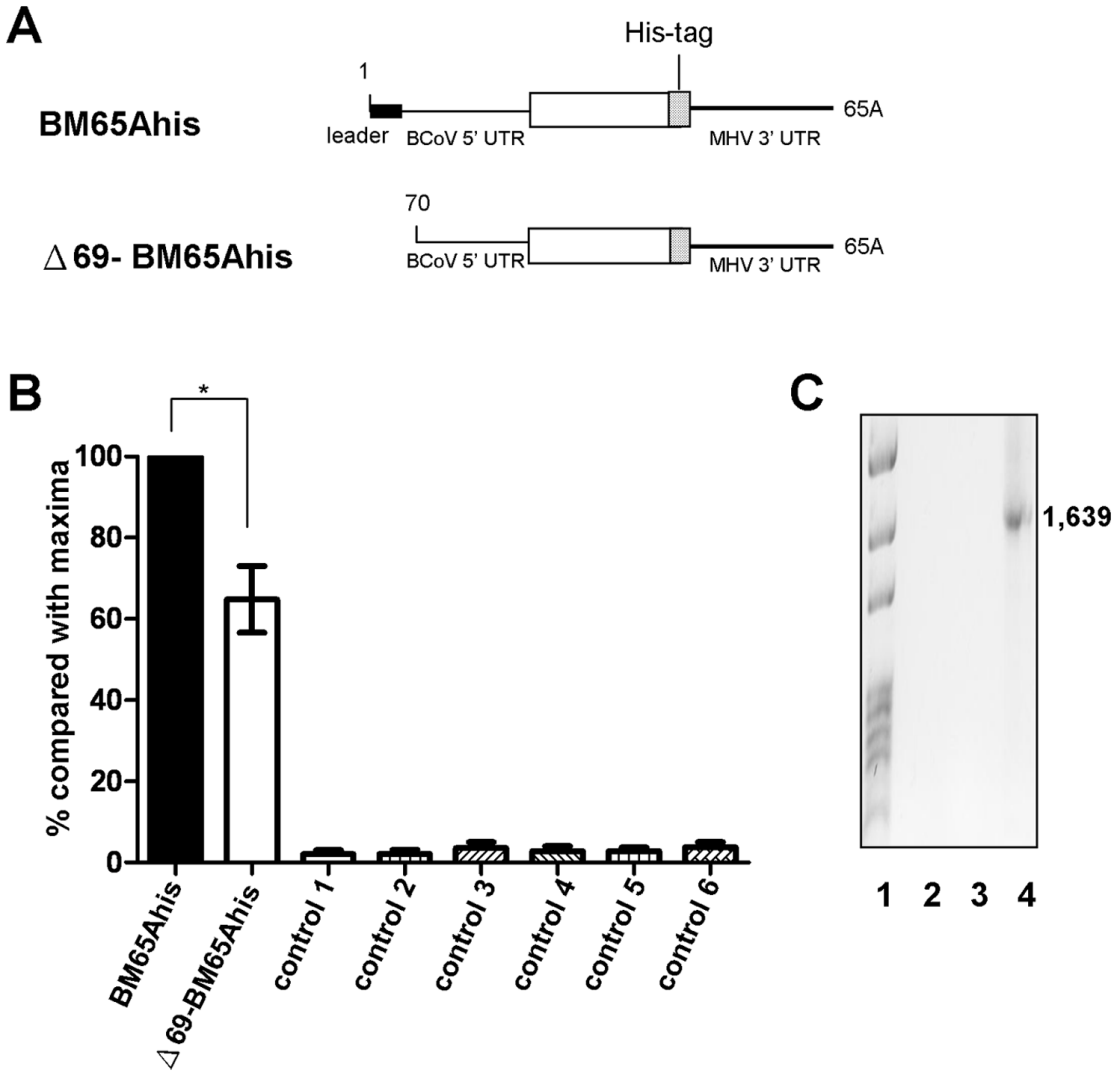
Effect of leaderless DI RNA on negative-strand synthesis. (A) DI RNA constructs used to test the efficiency of negative-strand DI RNA synthesis. (B) Quantitation analysis of negative-strand DI RNA synthesis, as measured by qRT-PCR. BCoV-infected HRT cells at 2 hpi were transfected with the indicated DI RNA, and total cellular RNA was extracted at 8 hpt to determine the efficiency of negative-strand synthesis for BM65Ahis and Δ69-BM65Ahis. Controls for qRT-PCR: control 1, total cellular RNA from mock-infected cells; control 2, total cellular RNA from BCoV-infected cells; control 3, total cellular RNA from BM65Ahis -transfected mock-infected cells; control 4, total cellular RNA from Δ69-BM65Ahis -transfected mock-infected cells; control 5, a mixture of BCoV-infected cellular RNA extracted at 8 hpt and BM65Ahis transcript; control 6, a mixture of BCoV-infected cellular RNA extracted at 8 hpt and Δ69-BM65Ahis transcript. (C) RT-PCR to detect potential recombination between the BCoV genome and DI RNA. The same strategy described in Fig. 2E and 2H was used here for the detection of potential recombination between the BCoV genome and BM65Ahis (Fig. 3C, lane 2) or Δ69-BM65Ahis (Fig. 3C, lane 3). A recombinant DNA of 1,639 nt was produced to serve as a size marker, as described for Fig. 2E and 2H. The values (B) represent the mean±SEM of three individual experiments. *p<0.05.

Following translation, the first step in coronavirus replication is synthesis of the negative-strand copy of the genome that in turn serves as a template for the synthesis of the genomic RNA positive strand. The coronavirus leader sequence has been demonstrated to be a *cis*-acting element critical for BCoV DI RNA replication [19]. Although the role of the leader sequence in BCoV DI RNA negative-strand RNA synthesis has not been identified, it has been suggested that it is not required in (−)-strand synthesis of MHV DI RNA using the CAT gene as a reporter [20]. To determine the requirement of the leader sequence in BCoV DI RNA for negative-strand RNA synthesis, the same BCoV DI RNA constructs (Fig. 3A), BM65Ahis (with the leader sequence) and Δ69-BM65Ahis (without the leader sequence) containing the MHV 3′ UTR, created for the translation assay shown in Fig. 2A were used. A head-to-tail ligation method along with RT-PCR has previously been used for detecting negative-strand BCoV DI RNA [45], [49]. In the present study, using head-to-tail ligation and qRT-PCR, the negative-strand counterparts from both the leaderless and leader-containing BCoV DI RNAs were also detected, although the efficiency from Δ69-BM65Ahis was ∼30% less than that from BM65Ahis (Fig. 3B). Furthermore, the negative-strand DI RNA detected was not from a potential DI RNA-coronavirus genome recombinant because no RT-PCR product derived from the recombinant was observed (Fig. 3C, lanes 2–3) with the primers used in Figs. 2E and 2H [49]. Therefore, these results suggest that the leader sequence is required for efficient negative-strand BCoV DI RNA synthesis.

### The Transcription Efficiency of DI RNA without the Leader Sequence is Impaired Compared with that of DI RNA with the Leader Sequence

**Figure 4:**
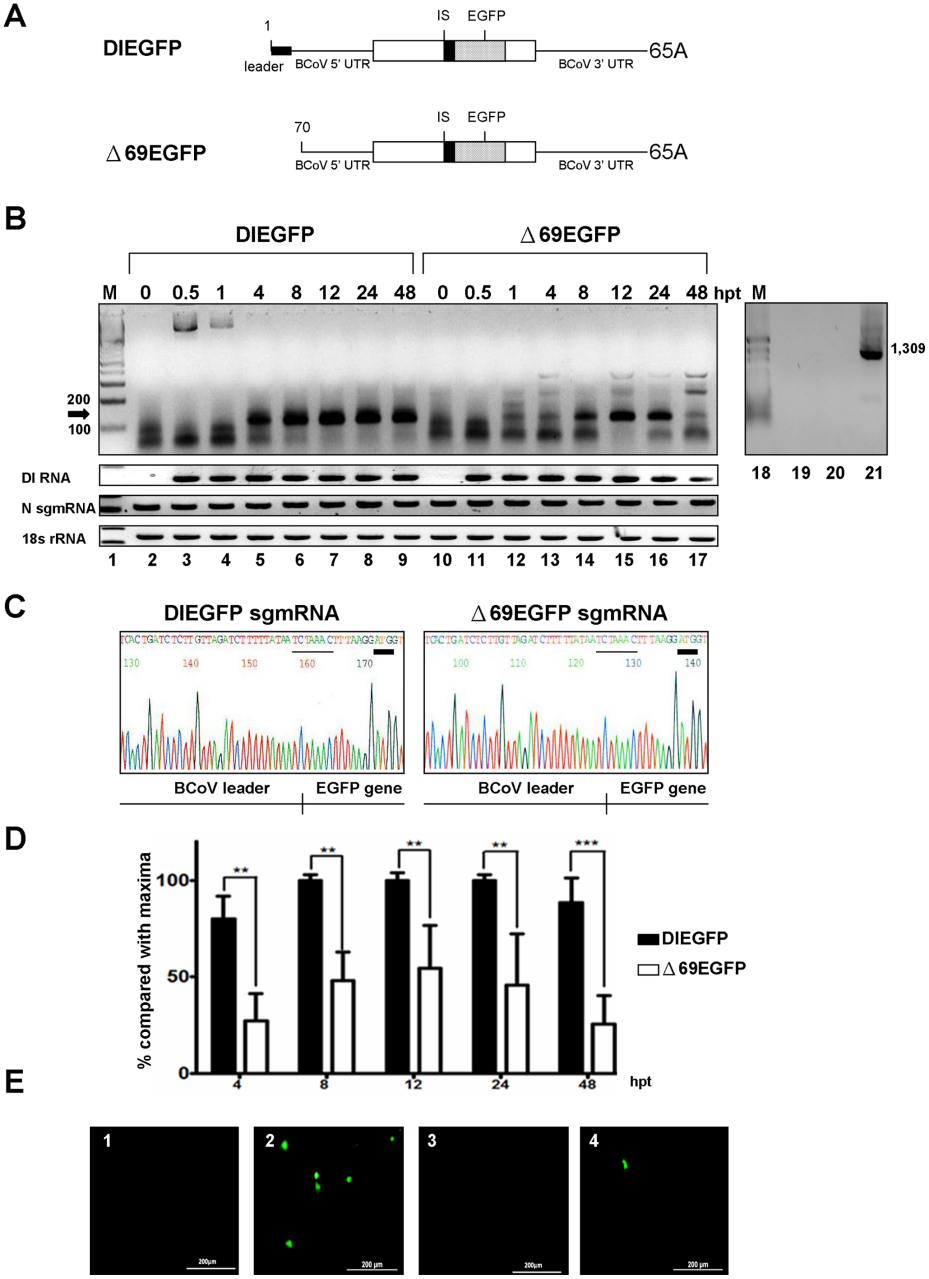
Effect of coronaviral leaderless DI RNA on transcription. (A) Constructs of DI RNA with the insertion of the intergenic sequence (IS) followed by the EGFP gene to test the effect of leaderless DI RNA on coronavirus transcription. (B) RT-PCR products with a length of 120 nt were observed from DIEGFP- (lanes 5–9, arrowhead) or Δ69EGFP- (lanes 12–17, arrowhead) transfected BCoV-infected cells. 18S rRNA, DI RNA, and helper virus N sgmRNA were used as internal controls. RNA extracted at 0 hpt was from mock-transfected BCoV-infected HRT-18 cells. To detect a potential recombinant between the BCoV genome and input DI RNA, the primers EGFP1(+), which anneals to the EGFP sequence and was used for RT, and M3(−), which anneals to the BCoV M protein gene, were used for PCR to detect potential recombination between the BCoV genome and DIEGFP (lane19) or Δ69EGFP (lane 20). A recombinant DNA of 1,309 nt (lane 21) was produced by overlap RT-PCR and was used as a size marker for the product generated using the primers EGFP1(+) and M3(−). (C) Sequence of the cDNA-cloned 120-nt RT-PCR product from Fig. 4B, lane 7 (left panel) and lane 15 (right panel) showing the leader-body junction (indicated with vertical bar), the IS UCUAAAC (indicated with thin line), and the AUG translation start codon (indicated with thick line) for EGFP. (D) Quantitation analysis of the 120-nt RT-PCR products from the individual DI RNA constructs shown in Fig. 4B. The efficiency of sgmRNA synthesis was normalized to the levels of the internal controls including 18S rRNA, DI RNA, and helper virus N sgmRNA. (E) Fluorescence of EGFP expressed from DIEGFP- or Δ69EGFP-derived sgmRNA. Panels 1 and 3 are mock-infected cells transfected with DIEGFP and Δ69EGFP, respectively; panels 2 and 4 are BCoV-infected cells transfected with DIEGFP and Δ69EGFP, respectively. In all cases, the cells were examined for fluorescence at 24 hpt. **p<0.01, ***p<0.001.

During coronavirus infection, a nested set of sgmRNAs are produced from which the coronavirus structural proteins are translated; these structural proteins have been linked to viral assembly and pathogenesis. To test whether the efficiency of sgmRNA synthesis is modulated by the leaderless genome as a template during infection, the intergenic sequence (IS), a transcription signal required for sgmRNA synthesis [41], [49], [50]–[51], and EGFP gene were engineered into BCoV DI RNA to create the constructs DIEGFP (with the leader sequence) and Δ69EGFP (without the leader sequence) (Fig. 4A) [49]. After transfection of DIEGFP into BCoV-infected HRT-18 cells, a 120-nt RT-PCR product was obtained using a primer set that anneals to the leader sequence and EGFP sequence; this product first appeared at 4 hpt and was abundantly present throughout the 48-h period of the experiment (Fig. 4B, lanes 5–9, arrowhead). Sequence analysis of the cloned 120-nt molecule obtained at 12 hpt confirmed the synthesis of sgmRNA as having come from DIEGFP, based on the leader-body junction sequence (Fig. 4C, left panel). A 120-nt RT-PCR product was also obtained with the same primer set from BCoV-infected HRT-18 cells transfected with Δ69EGFP. This product first appeared at 1 hpt and was present throughout the 48-h experiment (Fig. 4B, lanes 12–17). Sequence analysis revealed the leader-body junction site in the cloned 120-nt RT-PCR product, confirming that the observed sgmRNA was synthesized from Δ69EGFP (Fig. 4C, right panel). To confirm that the sgmRNA RT-PCR product was derived from DI RNA constructs rather than a recombinant between the genome and DI RNA as described above in Figs. 2E, 2H, and 3C, primers annealing to the BCoV M protein gene and DI RNA EGFP gene (for RT) were used in RT-PCR to detected a potential recombined product [49]. No RT-PCR product was identified (Fig. 4B, lanes 19–20) indicating that the synthesized sgmRNAs were from the input DI RNAs and not from a recombinant. After three rounds of independent experiments, the efficiency of sgmRNA synthesis from DIEGFP and Δ69EGFP was roughly quantitated (Fig. 4D) according to the intensity of the RT-PCR products and was normalized with the levels of internal controls at the same time point to ensure the samples were compared under the same conditions. The internal controls included DI RNA DIEGFP or Δ69EGFP, helper virus N sgmRNA, and 18S rRNA (Fig. 4B). As shown in Fig. 4D, the efficiency of sgmRNA synthesis from Δ69EGFP was less than that from DIEGFP during 4 h to 48 h of infection. Taken together, the results suggest that a leaderless genome carrying a transcription signal is able to serve as a template for sgmRNA synthesis; however, sgmRNA synthesis from the leaderless genome (Δ69EGFP) is less efficient than that from the leader-containing genome (DIEGFP).

To ascertain whether the encoded sgmRNA from the leaderless genome is translated, fluorescing HRT-18 cells were obtained as evidence of the expression of EGFP-containing sgmRNA. With respect to the upstream ORFs the EGFP ORF in both the Δ69EGFP and DIEGFP templates is in the −1 reading frame; therefore, EGFP expression from a fusion protein was not expected and the fluorescence observed from HRT-18 cells could only have resulted from expression of the encoded EGFP-containing sgmRNA. As predicted, EGFP fluorescence was observed in BCoV-infected HRT-18 cells transfected with either DIEGFP or Δ69EGFP (Fig. 4E, panels 2 and 4) but not in DIEGFP- or Δ69EGFP-transfected mock-infected HRT-18 cells (Fig. 4E, panels 1 and 3). The percentage of fluorescing cells was ∼0.8% for DIEGFP and ∼0.19% for Δ69EGFP. Together, the results suggest that the sgmRNA synthesized from the leaderless genome is able to serve as a template for translation although the percentage of fluorescing cells observed from Δ69EGFP (∼0.19%) was less than that for DIEGFP (∼0.8%). This result is also consistent with sgmRNA synthesis quantitated by RT-PCR (Fig. 4D) in which synthesis of sgmRNA from the leaderless DI RNA was also diminished.

## Discussion

Synthesis of a 3′ co-terminal nested set of sgmRNAs is a common feature of members of the Nidovirales [9], [15], [52]–[53]. In addition to this feature, the 5′ end of all sgmRNAs in arteri- and coronaviruses possesses a common leader sequence derived from the 5′ terminus of the genomic RNA [2], [7]–[9], [15], [17], [54]. Although ronivirus GAV sgmRNAs and torovirus EToV sgmRNAs 3, 4, and 5 have been identified as lacking a leader sequence, leaderless genomic RNA has not been documented in all members of Nidovirales [5]–[6]. In this study, we found for the first time a leaderless genomic RNA in coronavirus-infected cells at a late stage of persistent coronavirus infection, and this finding prompted us to analyze the functional role of the leaderless genome in coronavirus infection.

**Figure 5:**
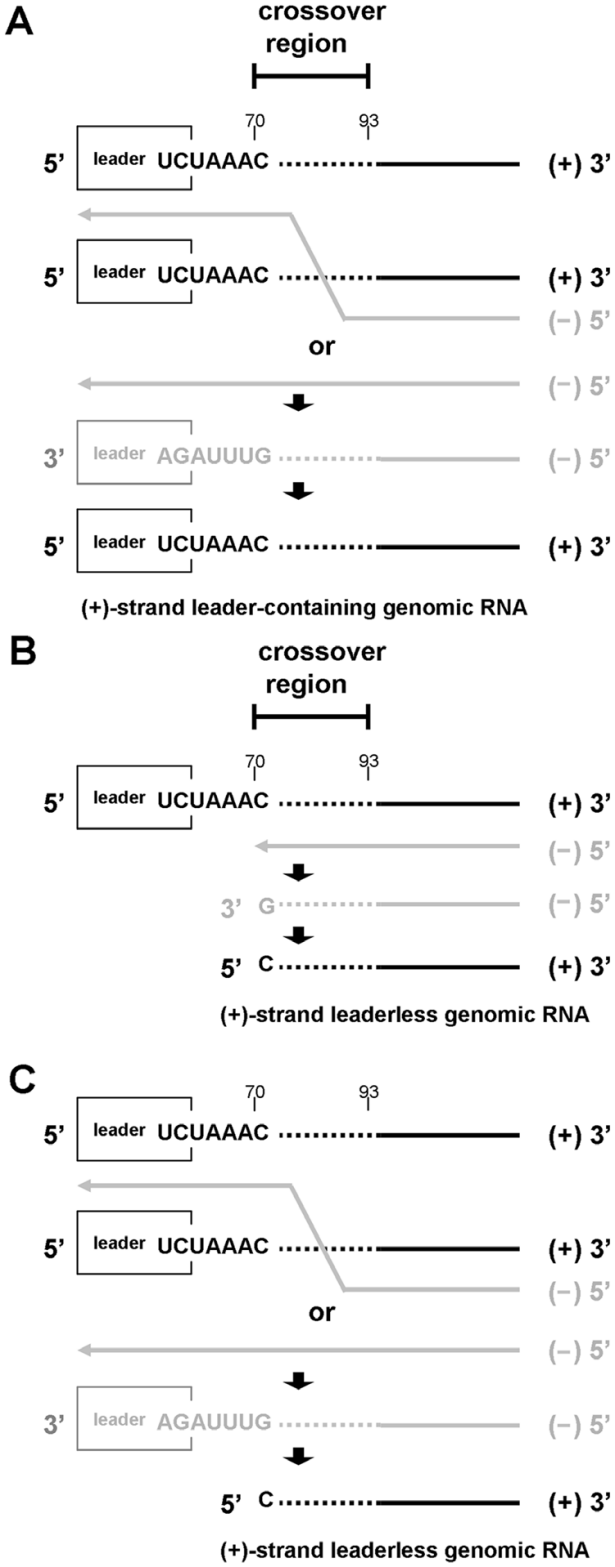
Schematic hypothetical model for the synthesis of leader-containing and leaderless genomic RNA during BCoV persistent infection. (A) Model for the synthesis of leader-containing genomic RNA. During negative-strand synthesis (indicated in gray) using positive-strand genomic RNA as a template (indicated in black), coronavirus polymerase switches template at the crossover region to acquire a leader sequence, generating negative-strand genomic RNA with the leader sequence. Alternatively, coronavirus polymerase may stay on the same positive-strand template to complete the synthesis of negative-strand genomic RNA containing a leader sequence. The synthesized negative-strand genomic RNA is then employed as a template to generate positive-strand genomic RNA with a leader sequence. The leader sequence is indicated with an open rectangle, and the polymerase crossover region is indicated with a dashed line. (B) Model for the synthesis of positive-strand leaderless genomic RNA using negative-strand leaderless genome as a template. Coronavirus polymerase employs positive-strand genomic RNA as a template to synthesize negative-strand genomic RNA. However, if template switching does not occur within the crossover region, the intergenic sequence on the positive-strand genomic RNA may serve as a terminator. Polymerase then falls off after copying the 3′-most nucleotide (cytosine) of the intergenic sequence UCUAAAC at position 70 on the positive-strand genomic RNA, which is also the 5′-most nucleotide of the crossover region on the positive-strand genomic RNA with respect to template switching during negative-strand synthesis, leading to the synthesis of leaderless negative-strand genomic RNA. The resulting leaderless negative-strand genomic RNA in turn serves as a template for the generation of leaderless positive-strand genomic RNA. (C) Model for the synthesis of positive-strand leaderless genomic RNA using negative-strand leader-containing genome as a template. After the synthesis of the leader-containing negative-strand genomic RNA, coronavirus polymerase recognizes the 3′-proximal intergenic sequence on the negative-strand leader-containing genomic template as a promoter and internally initiates the synthesis of positive-strand leaderless genomic RNA.

The RNA-dependent RNA polymerase (RdRp) template switching event that occurs during negative-strand synthesis to produce a negative-strand leader template on negative-strand sgmRNA molecules may yield a mechanistic explanation for synthesis of the leaderless genomic RNA. This notion is supported by the following previous findings. First, it has been suggested that, for equine torovirus sgmRNAs 3–5, the body intergenic sequences on the positive-strand genome sever as terminators of transcription during negative-strand synthesis. In turn, the intergenic sequences at the 3′ end of the negative-strand of sgmRNAs 3–5 are used as promoters for the synthesis of the sgmRNA positive strand resulting in the synthesis of leaderless sgmRNAs 3–5 [6], [55]. This model may also be applied for the synthesis of leaderless genomic RNA. Second, the leaderless genomic RNA observed in this study lacked the first 69 nt of the entire 5′ UTR sequence, which is 4 nt beyond the 65-nt leader. That is, the first nt of the 5′-end leaderless genomic RNA is the 70^th^ nt of the 5′-end leader-containing genomic RNA. Interestingly, the high-frequency crossover region of leader switching during negative-strand BCoV DI RNA synthesis is from the 70^th^ to 93^th^ nt [16]; that is, the 3′-most nucleotide of the crossover region on the negative-strand BCoV DI RNA in that study was the initiation site of the positive-strand leaderless genomic RNA in the present study. Accordingly, based on these findings, the intergenic sequence may play the dual roles of promoter and terminator [6], [55], and the 5′-most nt of the crossover region on the positive-strand BCoV DI RNA [16] is the 5′-terminal nt of the leaderless genomic RNA observed in this study. Therefore, we propose the following model for how the leader-containing and leaderless genomes are generated (Fig. 5). (1) During negative-strand genomic RNA synthesis using positive-strand genomic RNA as a template, polymerase strand switching would occur at any site within the crossover region to acquire the leader sequence. This would then lead to the synthesis of negative-strand leader-containing genomic RNA. The synthesized negative-strand leader-containing genomic RNA would in turn be employed as a template for the synthesis of positive-strand leader-containing genomic RNA (Fig. 5A). (2) If RdRp template switching does not occur, the intergenic sequence downstream of the leader sequence on the positive-strand genomic RNA would serve as a terminator, and the newly synthesized negative-strand genomic RNA would stops at the 70^th^ nt. The 70^th^ nt is a site located at the 5′-most crossover region with respect to the positive-strand genomic RNA and this process would result in a leaderless negative-strand genomic RNA for which the 3′-end 69-nt leader sequence is missing. The newly synthesized negative-strand leaderless genomic RNA would then serve as a template for the synthesis of a positive-strand leaderless genomic RNA (Fig. 5B). Although the negative-strand leaderless genomic RNA was not detectable in this study (Fig. 1F), the finding of the leaderless sgmRNA 7 negative strand which also lacked the 5′-terminal 69 nt (Fig. S1) provides further evidence in support of this model. Since the negative-strand leaderless genomic RNA was not identified in the current study (Fig. 1F), an alternative model could be that after synthesis of the leader-containing negative-strand genomic RNA during positive-strand RNA synthesis, the coronaviral polymerase recognizes the 3′-proximal intergenic sequence on the negative-strand leader-containing genomic template as a promoter and internally initiates synthesis of positive-strand leaderless genomic RNA (Fig. 5C). Further studies are required to elucidate a detailed mechanism of how the leaderless genome is synthesized during BCoV persistence.

The leaderless genomic RNA identified during BCoV persistent infection is not likely to be an artifact or a cleaved product because (1) the head-to-tail RT-PCR product of the leaderless genome was observed only at 67 and 97 dpi but not at other time points and (2) RT-PCR product with a length smaller than 200 bp was not observed when extracted RNA was not treated with tobacco acid pyrophosphatase as shown in Fig. 1E. In addition, head-to-tail ligation is performed at the viral RNA and not at the cDNA level. Therefore, even if a premature cDNA is produced the PCR product is not likely to be detected since the primers used anneal to both sides (5′ UTR and 3′UTR) of the poly(A) tail junction (Fig. 1A). Although the reason why the coronavirus leaderless positive-strand genomic RNA is not observed during acute infection is unknown, we speculate that since there is highly active translation taking place and the factors involved in template switching are in active that the overall process leads to the predominant synthesis of leader-containing RNA. The overwhelming abundance of leader-containing genomic RNA may subsequently lead to undetectable leaderless genomic RNA due to the feature of PCR competition [56]. In contrast, replication may be limited during coronavirus persistence [37], and the leaderless RNA can gradually be detected (Fig. 1B) because of the reduced number of leader-containing RNA molecules synthesized.

The discovery of a leaderless genome during coronavirus persistent infection in HRT-18 cells has led us to attempt a systematic analysis of leaderless genome behavior. It has been shown that substitution of the α-globin mRNA 5′ UTR with the MHV leader sequence enhances the translation of α-globin mRNA [22]. Consistent with this result, in the present study deletion of the leader sequence in BCoV DI RNA decreased the genome translation efficiency. In contrast to the results indicating that the MHV DI RNA leader sequence is nonessential for negative-strand synthesis, the BCoV DI RNA leader sequence was required for the efficient synthesis of its negative-strand counterpart in the present study. Additionally, different from the results in which the deletion of the sequence between nt 25 and 59 within the MHV DI RNA leader sequence almost blocked sgmRNA synthesis [21], the results of the present study suggest that the leaderless BCoV DI RNA was still able to synthesize sgmRNAs although the efficiency of sgmRNA synthesis from the BCoV leaderless DI RNA was ∼2- to ∼3- fold less than that from the leader-containing BCoV DI RNA during the 48 h of infection (Fig. 4D). Note that the decreased transcription efficiency from BCoV leaderless DI RNA at earlier times may attribute to the lack of leader sequence but not the number of DI RNA since the amount of the templates between input leader-containing and leaderless DI RNA was almost the same at 4 and 8 hpt as shown in Fig. 4B. However, in addition to the deletion of leader sequence, the less templates of leaderless DI RNA used for transcription could also be a factor to impair the efficiency of sgmRNA synthesis at later times of infection despite that the quantitated efficiency of transcription was normalized with the internal controls (Fig. 4B). The reasons for the various observations remain unclear; however, we speculate that the experimental approaches may be largely responsible for the different outcomes. Regardless, the leader sequence on genomic RNA is undoubtedly a crucial *cis*-acting element required for translation, replication (including negative-strand synthesis), and transcription during infection using BCoV DI RNA as a test system.

With regard to the significance of the leaderless genomic RNA identified during coronavirus persistence, the 5′ UTR of many positive-strand RNA viruses has been suggested to function in translation, replication, and transcription [57]–[65], and alterations of critical elements in the 5′ UTR have been linked to the persistence of coronaviruses [23]–[24]. For BCoV, the attenuated sgmRNA translation efficiency during persistence is associated with 5′ intraleader mutations of sgmRNA molecules [23], whereas mutations in the 5′ UTR of the MHV genome enhance translation and the subsequent replication and transcription during persistence [24]. Because functional analyses have revealed that the leader sequence is critical for translation, replication, and transcription of the viral genome in BCoV, as determined in the present study, we propose that the significance of leaderless genomic RNA identified during coronavirus persistence is to restrict the expression of viral genes, leading to reduced cytopathic effects, and ultimately may be associated with the establishment of persistent infection. As mentioned above, the selection of leaders with an intraleader ORF in BCoV sgmRNAs has been indicated as a mechanism to maintain BCoV persistence [23]. Therefore, in addition to the finding of leaderless genomic RNA in the present study, at least two structural alterations in the 5′ UTR of the genome and sgmRNA have been employed by BCoV to attenuate the gene expression that may correlate to the persistent infection of BCoV.

In this study, we discovered previously unidentified leaderless genomic RNA during BCoV persistent infection in cell culture, providing additional evidence that the members of Nidovirales may share a common ancestor. Functional analyses of the leaderless genomic RNA using BCoV DI RNA revealed that the leader sequence on the genomic RNA is required for coronaviral translation, replication, and transcription. Whether the leaderless genome is selected and contributes to the establishment of persistence needs to be further determined. Many aspects deriving from this study also need to be resolved. (1) Is leaderless sgmRNA synthesized during coronavirus persistent infection in addition to the leaderless genome discovered in this study? (2) Are there other mutations or structural changes in the BCoV genome or subgenome related to the maintenance of BCoV persistent infection? (3) Are there sequence alterations selected in the replicase gene that may be associated with the synthesis of leaderless genomic RNA? The elucidation of these questions will contribute to the further understanding of coronavirus persistence.

## Materials and Methods

### Establishment of Persistently BCoV-infected HRT Cells

A DI RNA-free stock of the Mebus strain of BCoV (GenBank accession no. U00735) was plaque-purified three times and grown in a HRT-18 cell line, as described [54], [66]–[67]. Persistently BCoV-infected HRT-18 cells were established by infection at a multiplicity of infection (MOI) of 5. After acute infection, the surviving cells (∼10%) were passaged at every fourth day thereafter. Total cellular RNA was extracted with TRIzol (Invitrogen) during acute and persistent infection.

### Head-to-tail Ligation of Viral RNA to Determine the Terminal Sequence of Genomic RNA

A head-to-tail ligation method has previously been used to identify the terminal features of the RNA genome [49], [68]. To determine the terminal sequence of viral positive-strand genomic RNA during BCoV persistence, RNA population with the poly(A) tail was enriched from a 20-µg sample of extracted total cellular RNA by Sera-Mag Oligo(dT)-coated Magnetic Particles (Thermo Scientific), treated with alkaline phosphatase (New England Biolabs) and extracted with phenol-chloroform. The extracted RNA in 25 µl of water was combined with 3 µl of 10X buffer and 10 U of (in 1 µl) tobacco acid pyrophosphatase (Epicentre) to de-block the 5′ capped end of the RNA. Following decapping, RNA was extracted with phenol-chloroform. The extracted RNA in 25 µl of water, 3 µl of 10X ligase buffer, and 2 U (in 2 µl) of T4 RNA ligase I (New England Biolabs) were combined, and the mixture was incubated for 16 h at 16°C. To determine whether or not the leaderless genome is a degradation product, the RNA sample was also prepared as described above but without treatment of alkaline phosphatase and tobacco acid pyrophosphatase. The ligated RNA was Phenol-chloroform-extracted and used for the RT reaction with SuperScript III reverse transcriptase (Invitrogen) and PCR with AccuPrime Taq DNA polymerase (Invitrogen). To determine the 5′-terminal features of the positive-strand genomic RNA, primer 2: BCV107(+), which binds nt 107–129 from the 5′ end of the BCoV positive-strand, was used for RT; for PCR, 5 µl of the resulting cDNA mixture was used in a 50-µl reaction with primer 2: BCV107(+) and primer 1: BCV3′UTR1(−), which anneal to nt 47–70 from the poly(U) tail on the negative-strand of the BCoV 3′ UTR. To determine the terminal sequence of viral negative-strand genomic RNA and sgmRNA, total cellular RNA was treated with tobacco acid pyrophosphatase (Epicentre), ligated with T4 RNA ligase I (New England Biolabs) and primer 1: BCV3′UTR1(−) was used for RT; for PCR, primers BCV3′UTR(−) and BCV107(+), and primers BCV3′UTR(−) and RYN(+) were used for determining terminal sequence of negative-strand genomic RNA and subgenomic mRNA, respectively. The resulting 50-µl PCR mixture was heated to 94°C for 2 min and subjected to 50 cycles of 30 s at 94°C, 30 s at 55°C, and 30 s at 72°C. The predicted PCR product was sequenced to determine the 5′-terminal features of the positive-strand genomic RNA. All primers used in this study are listed in Table S1.

### Plasmid Constructs

Construction of pBM65Ahis has been described previously [19], [49]. To generate the pΔ69-BM65Ahis construct, an overlap PCR was employed to delete the first 69 nt of BCoV 5′ UTR [51]. For this, oligonucleotides pGEMNDEI(−) and NL(+) were used with pBM65A DNA in the first PCR, and oligonucleotides NL(−) and RYN(+) were used with pBM65A DNA in the second PCR. Oligonucleotides pGEMNDEI(−) and RYN(+) were used with the products of the first two reactions in a third PCR to amplify a 1235-nt product that was cloned into the TOPO XL vector (Invitrogen). From this, a 933-nt fragment obtained by digestion with *NgoM*IV and *Xba*I was cloned into *NgoM*IV- and *Xba*I-linearized psBM65A to produce construct pΔ69-BM65Ahis. To generate pBM65AhisΔ5, oligonucleotides TGEV 7(−) and Δ5(+) were used with pBM65Ahis DNA in the first PCR, and oligonucleotides Δ5(−) and DI reverse(+) were used with pBM65Ahis DNA in the second PCR; oligonucleotides TGEV 7(−) and DI reverse(+) were used in a third PCR with the products of the first two reactions to amplify a 1248-nt product that was cloned into the TOPO XL vector (Invitrogen). Next, an 801-nt fragment obtained by digestion with *Spe*I and *Mlu*I was cloned into *Spe*I- and *Mlu*I-linearized pBM65Ahis to produce the pBM65AhisΔ5 mutant. To construct pΔ69-BM65AhisΔ5, an 801-nt fragment obtained by digestion of pBM65AhisΔ5 with *Spe*I and *Mlu*I was cloned into *Spe*I- and *Mlu*I-linearized pΔ69-BM65Ahis to produce the pΔ69-BM65AhisΔ5 mutant. Construct pDIEGFP, formally called pDIRNA-3, with the template-switching signal (intergenic sequence; IS) and EGFP gene was previously described [49]. To generate construct pΔ69EGFP, pD69-BM65Ahis was digested with *Xba*I- and *Ngom*IV, and the digested fragment carrying a 69-nt deletion of the BCoV 5′ UTR was cloned into *Xba*I- and *Ngom*IV -linearized pDIEGFP.

### Western Blot Analysis for *in vivo* DI RNA Translation

The DNA constructs were linearized with *Mlu*I, transcribed *in vitro* with the mMessage mMachine T7 transcription kit (Ambion) according to the manufacturer’s instructions, and passed through a Biospin 6 column (Bio-Rad), followed by transfection [41]. For transfection, HRT-18 cells in 35-mm dishes at ∼80% confluency (∼8×10^5^ cells/dish) were infected with BCoV at a multiplicity of infection of 5 PFU per cell and transfected 2 h later with 3 µg of transcript RNA using Lipofectin (Invitrogen) [19]. After transfection, proteins from HRT-18 cell lysates were harvested, electrophoresed through 12% SDS-PAGE gels, and electrotransferred to nitrocellulose membranes (Amersham Biosciences). DI RNA fusion proteins were detected using an antibody specific to the histidine tag or β-actin (Serotec) as the primary antibody and goat anti-mouse IgG conjugated to HRPO as the secondary antibody (Jackson Laboratory). The proteins detected were visualized using Western Lightning™ Chemiluminescence Reagent (Perkin Elmer NEL105) and X-ray film (Kodak) [69].

### Real-Time RT-PCR Analysis of Negative-strand DI RNA Synthesis

To analyze the synthesis efficiency of negative-strand DI RNA [49], HRT-18 cells in 35-mm dishes at ∼80% confluency were infected with BCoV at a multiplicity of infection of 5 PFU per cell. After 2 h of infection, 3 µg of DI RNA transcript was transfected into the BCoV-infected HRT cells; total cellular RNA was extracted with TRIzol (Invitrogen) at 8 hpt. A 10-µg sample of extracted total cellular RNA in 25 µl of water was combined with 3 µl of 10X buffer and 10 U of (in 1 µl) tobacco acid pyrophosphatase (Epicentre) to de-block the 5′ capped end of the RNA. Following decapping, RNA was extracted with phenol-chloroform, heat-denatured at 95°C for 5 min, and then quick-cooled. A 3-µl aliquot of 10X ligase buffer and 2 U (in 2 µl) of T4 RNA ligase I (New England Biolabs) were added, and the mixture was incubated for 16 h at 16°C. After ligation, RNA was phenol-chloroform-extracted and quantitated, and 1 µg of ligated RNA was used for an RT reaction to synthesize cDNA with SuperScript III reverse transcriptase (Invitrogen). The real-time PCR amplification was performed using TaqMan®Universal PCR Master Mix (Applied Biosystems) according to the manufacturer recommendations with a LightCycler® 480 instrument (Roche Applied Science) and primers MHV6(−) and BCV23-40(+) (Table S1). To quantitate the synthesis of negative-strand DI RNA, dilutions of plasmids containing the same gene as the detected RT-PCR product of the negative-strand DI RNA were always run in parallel with the quantitated cDNA for use as standard curves (the dilutions ranged from 10^8^ to 10 copies of each plasmid). The efficiency of the negative-strand RNA synthesis was normalized to the levels of the internal controls including 18S rRNA, DI RNA, and helper virus N sgmRNA; the primers used for these internal controls are described in Table S1. The reactions included an initial pre-incubation at 95°C for 5 min, followed by 45 amplification cycles of 95°C for 15 s and 60°C for 60 s.

### Northern Assay for DI RNA Replication

HRT-18 cells in 35-mm dishes at ∼80% confluency (∼8×10^5^ cells/dish) were infected with BCoV at a multiplicity of infection of 5 PFU per cell. After 2 h of infection, 3 µg of transcript were transfected into the BCoV-infected HRT-18 cells. The supernatant was harvested at 48 hpt, and 500 µl was used to infect freshly confluent HRT-18 cells in a 35-mm dish (virus passage 1, VP1). Total cellular RNA was extracted with TRIzol (Invitrogen) at 48 hpi (VP1), and 10 µg of total RNA was electrophoresed through a formaldehyde-agarose gel. RNA was transferred from the gel to a Nytran membrane by vacuum blotting, and the blots were probed with the TGEV8(+) oligonucleotide, which was 5′-end labeled with ^32^P. The probed blot was exposed to Kodak XAR-5 film for 16 h at −80°C. For quantitating DI RNA replication, the probed blots were analyzed using a Packard InstantImager Autoradiography System.

### Detection of DI RNA-derived sgmRNA

The transcripts used for the detection of sgmRNA from DI RNA were synthesized from *Mlu*I-linearized pDIEGFP and pD69EGFP *in vitro* with the mMessage mMachine T7 transcription kit (Ambion) according to the manufacturer’s instructions. For transfection, HRT-18 cells in 35-mm dishes at ∼80% confluency (∼8×10^5^ cells/dish) were infected with BCoV at a multiplicity of infection of 5 PFU per cell and then transfected 2 h later with 3 µg of transcript using Lipofectin (Invitrogen) [19]. For detection of EGFP expressed from sgmRNA, mock-infected or BCoV-infected HRT-18 cells transfected with DIEGFP and Δ69EGFP at 24 hpt were subject to EGFP determination using fluorescence microscopy (Olympus, Japan). For detection of sgmRNA derived from DI RNA, total RNA was extracted from HRT-18 cells using TRIzol (Invitrogen) at the time points indicated in Fig. 4B. A 5-µg sample of extracted total cellular RNA was reversed transcribed with SuperScript III reverse transcriptase (Invitrogen) and oligonucleotide EGFP2 (+), which is complementary to the positive strand of the EGFP gene. The PCR reactions were performed with AccuPrime Tag DNA polymerase (Invitrogen) and oligonucleotides leader20(−), which is complementary to the negative-strand leader sequence, and EGFP1(+), which is complementary to the positive strand of the EGFP gene; the conditions were 94°C for 2 min and 29 cycles of 30 s at 94°C, 30 s at 55°C, and 30 s at 72°C. The ∼150-bp PCR products were gel-purified, cloned into TOPO XL PCR (Invitrogen), and sequenced.

## Supporting Information

Figure S1 **Identification of negative-strand leaderless sgmRNA 7 during BCoV persistent infection.** (A) Total cellular RNA extracted from BCoV-persistently infected cells was treated with tobacco acid pyrophosphatase and ligated with T4 RNA ligase I. The RT-PCR product was synthesized with primers BCV3′UTR1(−) (for RT) and RYN(+). RT-PCR products with a larger size of ∼300 bp (lanes 2–7, marked with black arrowhead) and with a smaller size of ∼200 bp (lanes 5–7, marked with white arrowhead) were observed. (B) The upper panel shows part of the first 88-nt sequence of the 5′ UTR in the negative-strand BCoV sgmRNA 7. The positions (1 and 70) are given on the top of the sequence, and the intergenic sequence (IS) AGAUUUG is underlined. The lower panel shows the sequence (shown in the negative strand) of the cDNA-cloned RT-PCR product with a size of ∼300 bp from lane 7, as indicated with a black arrowhead in Fig. S1A. (C) The upper panel shows the sequence of the 5′UTR on the negative-strand BCoV sgmRNA 7, which lacks the first 69 nts; position 70 is given on the top of the sequence. The lower panel shows the sequence (shown in the negative strand) of the cDNA-cloned RT-PCR product with a size of ∼200 bp from lane 7, as indicated with a white arrowhead in Fig. S1A. M, ds DNA size markers in nt pairs. dpi: days postinfection.
(TIF)

Table S1 **Oligonucleotides used for this study.**
(TIF)
