# Supplementary figures and images for: A Leaderless Genome Identified during Persistent Bovine Coronavirus Infection Is Associated with Attenuation of Gene Expression
Source: PLoS One. 2013 Dec 12;8(12):e82176. doi: 10.1371/journal.pone.0082176 (PMC3861326; doi:10.1371/journal.pone.0082176)

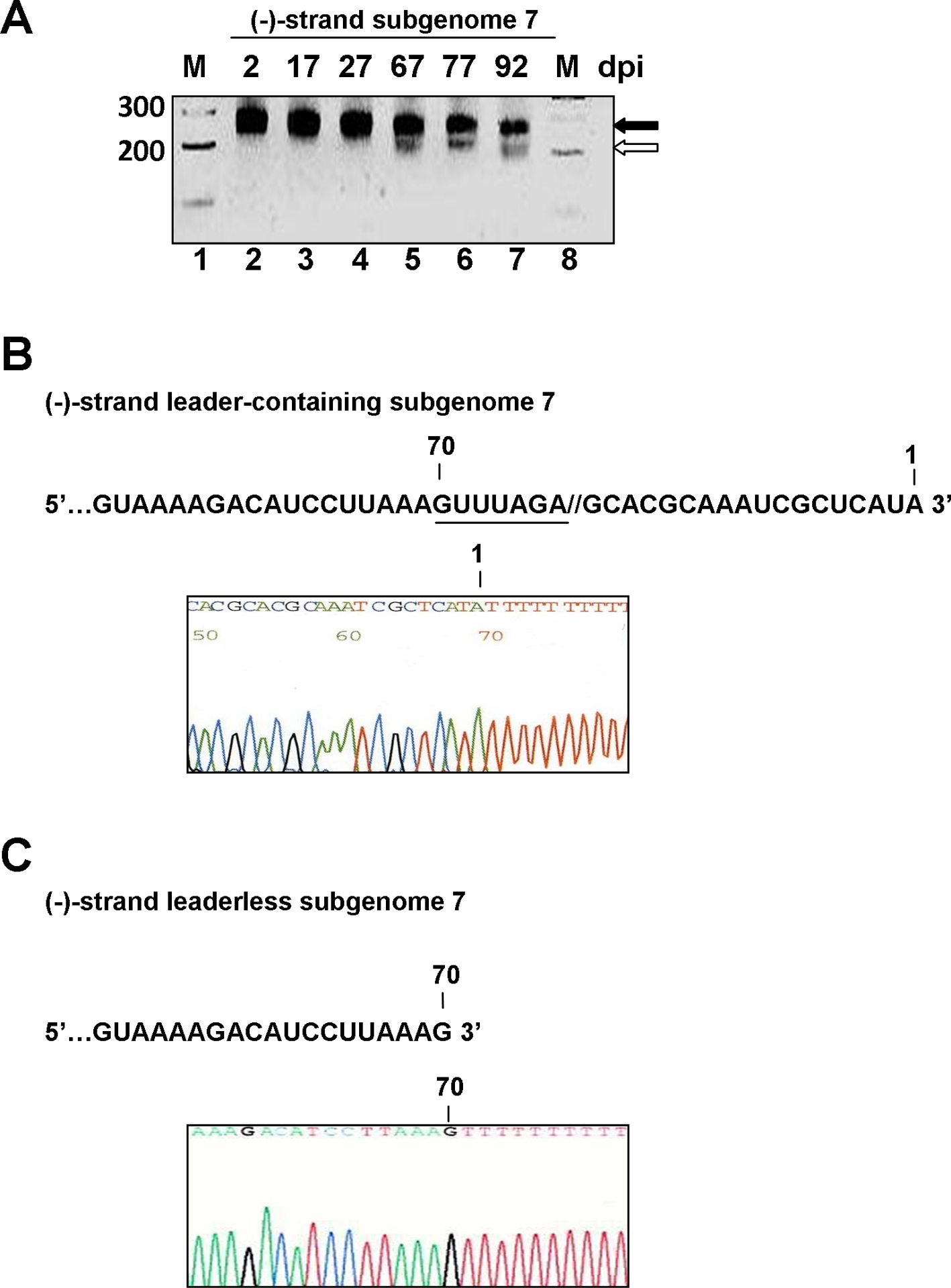

Supplement: Figure S1 — Identification of negative-strand leaderless sgmRNA 7 during BCoV persistent infection. (A) Total cellular RNA extracted from BCoV-persistently infected cells was treated with tobacco acid pyrophosphatase and ligated with T4 RNA ligase I. The RT-PCR product was synthesized with primers BCV3′UTR1(−) (for RT) and RYN(+). RT-PCR products with a larger size of ∼300 bp (lanes 2–7, marked with black arrowhead) and with a smaller size of ∼200 bp (lanes 5–7, marked with white arrowhead) were observed. (B) The upper panel shows part of the first 88-nt sequence of the 5′ UTR in the negative-strand BCoV sgmRNA 7. The positions (1 and 70) are given on the top of the sequence, and the intergenic sequence (IS) AGAUUUG is underlined. The lower panel shows the sequence (shown in the negative strand) of the cDNA-cloned RT-PCR product with a size of ∼300 bp from lane 7, as indicated with a black arrowhead in Fig. S1A. (C) The upper panel shows the sequence of the 5′UTR on the negative-strand BCoV sgmRNA 7, which lacks the first 69 nts; position 70 is given on the top of the sequence. The lower panel shows the sequence (shown in the negative strand) of the cDNA-cloned RT-PCR product with a size of ∼200 bp from lane 7, as indicated with a white arrowhead in Fig. S1A. M, ds DNA size markers in nt pairs. dpi: days postinfection. (TIF) [file pone.0082176.s001.tif]
